# Patient experiences of behavioural therapy for bipolar depression: A qualitative study

**DOI:** 10.1111/bjc.12515

**Published:** 2024-12-10

**Authors:** Sakir Yilmaz, Anna Hancox, Molly Price, Jemma Regan, Barney Dunn, Heather O'Mahen, Kim Wright

**Affiliations:** ^1^ Department of Psychology Abdullah Gul University Kayseri Turkey; ^2^ Department of Psychology University of Exeter Exeter UK; ^3^ Unaffiliated; ^4^ University of Plymouth Greenbank UK; ^5^ Devon Partnership NHS Trust Exeter UK; ^6^ Mood Disorders Centre University of Exeter Exeter UK

**Keywords:** behavioural therapy, bipolar depression, patient experience

## Abstract

**Background:**

Although multiple qualitative studies have explored participants' experiences of behavioural activation (BA) for unipolar depression, none have investigated the experiences of BA in people with bipolar depression. This is of particular interest because qualitative studies concerning the experience of receiving therapy can help inform the theory of change underpinning the intervention.

**Aim:**

The purpose of this study was to explore the experiences and perspectives of individuals with bipolar disorder who received a course of one‐to‐one BA for bipolar depression. We sought to explore participants' experience of the effects of BA therapy, both proximally and distally.

**Method:**

Semi‐structured interviews were conducted with nine individuals meeting research diagnostic criteria for bipolar I or II disorder who had received up to 20 sessions of BA adapted for bipolar depression. Thematic analysis using a framework approach was used to explore and describe the experiences of participants.

**Results:**

Participants' perspectives on the impact of therapy were categorized under four subthemes: client behaviour inside and outside sessions, changes in clients' perspectives, the impact on symptoms and impact on life and functioning.

**Conclusions:**

Participants' accounts of the impact of therapy were broadly consistent with the theory underpinning a behavioural approach. Participants described a central role for perspective change, and particularly increased acceptance of the self and mood states, as facilitating behavioural changes and more distal benefits. Process evaluations embedded in future trials may include quantitative measures of key processes described by our participants, as well as those clearly implied by the behavioural theory of depression.

**Practitioner points**

- We carried out qualitative interviews with people who received behavioural therapy for bipolar depression.
- The way that participants described the effects of therapy aligned for the most part, with the theory underlying the behavioural approach.
- Practitioners could consider the potential role of fostering acceptance of self and condition as a facilitator of change.

## INTRODUCTION

Bipolar disorder (BD) is a well‐established, often recurrent, disorder characterized by the presence of manic or hypo‐manic and depressive episodes (Culpepper, 2014). It has been named as the fifth leading cause of disability amongst all mental health and substance use disorders (Ferrari et al., 2016). Although bipolar disorder is uniquely characterized by mania and hypomania, it is often characterized by depressive symptoms over the long term (Judd & Akiskal, 2003; Perlis et al., 2006). In addition, clients predominant experience is often depressive symptoms over the long term in between episodes of mania. To meet criteria for a depressive episode, a person must have experienced at least two weeks of low mood or loss of enjoyment plus symptoms such as reduced energy or a diminished ability to concentrate or think. Both bipolar and unipolar depression (UD: where no manic or hypomanic episodes are present) can be extremely challenging and debilitating conditions. The severity of depression in BD is significantly affected by the presence of other symptoms, such as manic or hypomanic episodes, which can have a significant influence on the overall course and severity of the disease (Swann et al., 2007). For example, the clinical characteristics of a bipolar episode were characterized by a greater number of shorter episodes of depression (Forty et al., 2008).

Evidence‐based pharmacological treatment options including lithium, anticonvulsants, antidepressants and atypical antipsychotics for bipolar depression are available. Treating depression in bipolar individuals can be particularly challenging due to the fact that some individuals may experience manic episodes in response to antidepressant medications, which may exacerbate the severity of the overall condition and result in an elevated risk of suicide (Ghaemi, 2008). For the purpose of preventing a switch to mania and antidepressant medications are typically combined with mood stabilizers or antipsychotic medications (Yatham et al., 2018).

There are a number of evidence‐based psychological therapies for UD and studies have examined these as standalone interventions of UD, in some cases in combination with antidepressant medication (Cuijpers et al., 2020). In recent decades, psychological approaches such as cognitive behavioural therapy, interpersonal and social rhythm therapy and family focussed intervention have been tested in relation to bipolar depression (Yilmaz et al., 2022). However, the small size and heterogeneity of the evidence base make it difficult to draw strong conclusions about the most effective approach. In addition to the need for effective psychological therapies for bipolar depression, the efficiency of the approach is also important. Efficiency refers to the effectiveness of a treatment in achieving positive results whilst considering factors such as cost, duration and ease of implementation. Given the prevalence of BD and its typically recurrent course, efficiency will be achieved in part by having a therapy protocol that is easily trainable amongst healthcare staff, meaning that it can be delivered by multiple groups of health professionals and without the need for extensive (and thus expensive) training.

A behavioural activation (BA) approach may be one such efficient approach. It aims to improve mood by decreasing avoidance of feared punishing consequences and supporting re‐engagement with meaningful and rewarding activities (Dimidjian et al., 2011). As such, standalone BA may be worthy of investigation in the treatment of bipolar depression. The effectiveness of behavioural treatments for UD has been comprehensively documented in numerous meta‐analyses (Ciharova et al., 2021; Cuijpers et al., 2021), and it has been argued that BA is relatively simple to deliver (Richards et al., 2016). Its working mechanism, however, it is little understood. In a systematic review, Janssen et al. (2021) examined the mediators of behavioural activation in relation to depression. Several factors prevented a definitive conclusion regarding whether any of the mediators were significant, including an insufficient degree of methodological quality in some studies, and variations in the questionnaires used.

To date, a small number of studies have investigated standalone BA as a treatment for bipolar depression. Weinstock et al. (2016) conducted a proof‐of‐concept trial of modified BA as an adjunctive treatment for bipolar depression to assess its preliminary feasibility and acceptability in hospital settings. In a 20‐week open trial, 16 therapy sessions were delivered to 12 participants who suffered from bipolar depression. The BA programme was found to be acceptable to patients, and there was a statistically significant improvement in depression symptoms in the sample overall from pre to post treatment. A more recent case series study has evaluated BA for bipolar depression in a U.K. outpatient setting, including recruitment from primary care and from the community (Wright et al., 2022). This study also found BA to be acceptable in terms of both patient attendance and their satisfaction ratings. The pattern of change across individual participants' scores on clinical outcome measures was consistent with the potential of the intervention to offer benefit. In both studies, it was considered necessary to modify the BA protocol used in UD, including the addition of psychoeducation about BD and work on detecting and managing early warning signs of (hypo)manic relapse, in addition to a focus on a sustainable pattern of activity that includes rests rather than maximizing rewarding activity. Modifying the BA protocol for BD entailed adapting the treatment to the disease's cyclical nature, addressing early signs of (hypo)manic relapse and promoting a balanced and sustainable activity pattern. Further development of BA for bipolar depression may benefit from an extended theoretical account of the mechanisms by which the intervention brings about clinical change.

Existing protocols are informed by the behavioural theory of the maintenance of unipolar depression outlined above and also by the corresponding theory of change; however, these accounts do not explicitly address the maintenance of excessive high mood, activation and approach tendencies, nor how behavioural concepts and techniques could help the individual to navigate these. Furthermore, it is important to distinguish between the theorized and actual mechanisms of change in complex interventions (Moore et al., 2015) understanding whether change does in fact occur via the pathways hypothesized or via other routes is important in order to refine and optimize the intervention and to train practitioners efficiently. Evaluation of the process of change within a complex intervention can be approached using quantitative and qualitative methods (Moore et al., 2015; Skivington et al., 2021), with qualitative methods potentially highlighting the priorities of patients within the treatment and suggesting potential mechanisms of action not previously considered. Whilst several qualitative studies have been conducted to explore participants experience of BA for UD, none have explored this with regard to bipolar depression.

Using qualitative data gathered from participants in the case series reported by Wright et al. (2022), the overall aim of the current study was to investigate participant's experience of BA in order to inform a behavioural therapy for bipolar depression. In particular, the main focus of the study was on perceived impact and mechanisms of change.

## METHOD

This study was conducted within a larger case series (Wright et al., 2022) evaluation, which gained appropriate ethical approval (HRA reference number 18/SW/0116) including for the qualitative aspect reported here. Participants gave written informed consent for both participation in the case series and separately for participation in the qualitative interviews themselves. Further details of the methods used in the case series are reported elsewhere (Wright et al., 2022).

### Participants

Participants were recruited from secondary care mental health services in a county in the south west of England as well as through advertisements in the local community between October 2018 and February 2020. All participants included in the case series were required to be at least 18 years old and suffering from a major depressive episode at the time of the study, according to DSM‐V criteria (5th ed.; DSM–5; American Psychiatric Association, 2013) assessed by a trained researcher using the SCID‐V (First et al., 2016). Additionally, participants were required to have (i) a score of >9 on a self‐reported depression severity measure the Patient Health questionnaire (PHQ‐9; Kroenke et al., 2001) and (ii) a DSM‐V diagnosis of bipolar I or II disorder, assessed using the SCID‐V. There was a requirement that participants were able to read and speak English to an extent that allowed them to make sufficient use of therapy and to complete the research assessments.

The following were exclusion criteria: (i) current or past learning disabilities, organic brain changes or substance dependence (drugs and alcohol) that would interfere with the ability to utilize psychotherapy; (ii) a marked risk to oneself that cannot be effectively managed within the context of an outpatient psychological therapy program; (iii) incapable of providing informed consent at the present time; (iv) presently undergoing other psychosocial treatment for depression or BD and (v) identification of another area of difficulty that should be the primary focus of the intervention, for example, post traumatic stress disorder.

A total of 12 participants entered the case series study. Of these, one withdrew from the study. Of the remaining 11, all completed therapy and were invited to take part in the interview. Nine agreed of the remaining two, one participant did not respond and the other declined to be interviewed.

### Intervention

The therapy consisted of upto 20 individual weekly therapy sessions as well as a booster session three months after the completion of therapy.

Sessions were delivered in‐person, however, during the latter part of the study, these were delivered by telephone or online video conferencing due to COVID‐19 restrictions. Two psychologists with specialist training in cognitive and behavioural therapies, including BA, conducted the therapy, one of whom had developed the therapy protocol.

The therapy was based on the behavioural activation protocol used in the treatment of unipolar depression as part of the COBRA trial (Richards et al., 2016). Adaptations were made to better meet the needs of patients with bipolar depression, including relationship to medication, and the extent to which the patient is able to access appropriate information about BD. Contracting work was carried out at an early stage with the patient in order to understand together how symptoms of hypomania/mania would be noticed and responded to within therapy, should these occur. Where useful, functional analysis (Virués‐Ortega & Haynes, 2005) was applied to instances of impulsive or mood‐driven behaviour in response to approach‐related affect (such as excitement or anger), in addition to instances of avoidance in response to negative affect. When planning in positive behaviours the scheduling process was adapted to (i) prioritize activities those that promote less ‘risky’ positive states rather than those that promote hypomania for that individual; (ii) strike a balance between rest and activity that supports the person's longer term well‐being and (iii) increase focus upon the potential role of daily routine in triggering and maintaining symptoms. Finally, relapse prevention planning was conducted with respect to manic as well as depressive relapse. Participants could opt to bring a friend or family member to one or more sessions if they wished, as per the original protocol.

### Procedure

After the first booster session or the final therapy session, if they did not opt for booster sessions, then each participant was invited to participate in a qualitative interview. Interviews lasted on average 45 min and were recorded, were conducted either face‐to‐face or over the telephone, occurred between September 2019 and November 2020, were around 1–3 months following the end of therapy, after the first booster session if taken up by the participant and were conducted by an assistant who assisted with the trial (JR) and a PhD student (SY).

We developed a semi‐structured topic guide based on the objectives of the study, as well as on previous research, (see Data S1) and with input from an individual with lived experience of BD. The participants were asked about the following: their overall views of the therapy, helpful and unhelpful aspects of the therapy, the impact of therapy and their views on treatment length and delivery format. Our questions were informed by key hypothesized areas of change in BA but also left space for participants to give their own views on how any change had happened. Verbatim transcripts were created by SY, a second member of the research team checked these for accuracy.

### Analysis

Thematic analysis using a framework approach was implemented to analyse the data. Thematic analysis is a flexible method for analysing qualitative data that involves searching across an entire data set in order to identify, analyse and report patterns that are repeating (Braun & Clarke, 2006). As a form of thematic analysis, framework analysis employs a predefined analytical framework to guide the interrogation of material and allow comparisons across participants. In our study, the approach taken was both deductive and inductive. Our topic guide and analytical framework were broadly reflective of a behavioural theory of change, in that change within the participant themselves (including their behaviours and thinking) was considered, as well as change within their context. At the same time, we allowed the framework to be guided by participant's reflections and observations; thus, we did not limit it to a particular set of expectations about the impact of therapy and how this was brought about.

Acquiring a comprehensive overview of all the data collected was the first step in the analysis. In order to familiarize themselves with the data, two researchers (SY and AH) read the transcripts multiple times. Preliminary analysis of four transcripts was conducted independently by the two researchers (SY and AH) with regular meetings to discuss potential codes, with input from a third researcher (KW). In the next step, a coding framework was agreed based upon both the codes observed to date and the key areas intended to be explored within interviews (acceptability of the therapy, impact and process of change). This was used by one researcher (SY) to code all transcripts according to the framework (indexing). During the indexing of transcripts, the framework was modified as necessary, in discussion with KW. The coded material was then analysed by SY to identify themes and develop a thematic framework. This was assisted by charting, whereby the quotes from participants were grouped according to theme or subtheme. During the final phase, a second researcher (AH) verified that the transcripts had been coded according to the thematic framework and discussed any differences in opinion with one researcher (SY).

To examine not only the subthemes but also the interrelationships between them, one researcher (SY) examined all transcripts and noted any instances where participants referred to relationships between two or more subthemes. This coding was checked by a second researcher (KW). This information was used to create a schematic depicting the interrelationships between subthemes as reported by participants (Figure 2). We used pseudonyms to protect participants' confidentiality.

Throughout the latter part of the analysis, the charting document was repeatedly referred to in order to ensure themes and subthemes accorded with direct quotations from participants. Production of a reflexive statements by the coders facilitated reflection upon their positionality in relation to the material being analysed.

## RESULTS

### Participant characteristics

Seven participants met research diagnostic criteria for bipolar I disorder, and the other two met criteria for bipolar II disorder. The age range of the nine participants at the time of interview was 33–65 years. All participants were female and white British. Four participants were employed outside the home, one was a homemaker, two were unemployed, and two were retired. All participants except one were prescribed psychiatric medication. At the start of the therapy period, according to their score on the PHQ‐9, one was experiencing moderate depression, five moderately severe depression and one severe depression, two participants had recovered from depression since study intake. By the end of the therapy period, the two participants who were recovered remained so. Six of the remaining seven participants recovered and showed reliable improvement according to their PHQ‐9 score, the seventh did not recover according to their PHQ‐9 score, nor did they show reliable change.

### Impact of therapy

We identified two superordinate themes from participant's reports: (1) the acceptability of therapy, (2) impact of therapy; here we focus upon the latter. Themes and example comments pertaining to acceptability are given in Data S2. Summary of themes and subthemes emerging from qualitative analysis is presented in Data S3.

**FIGURE 1:**
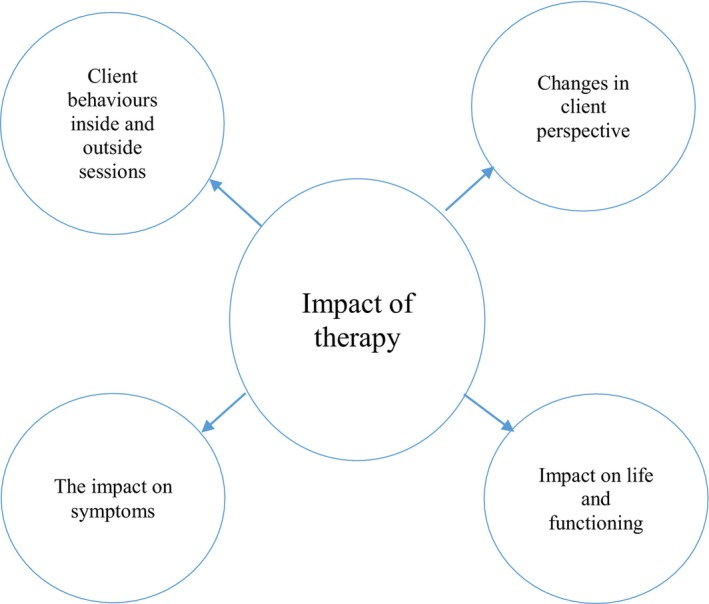
Schematic depicting of emerging theme and subthemes.

Under this theme, there were four subthemes that could be used to understand participants' opinions on how the therapy affected them, and the impact it had on their lives: client behaviours inside and outside sessions, changes in client perspective, the impact on symptoms and impact on life and functioning (Figure 1).

#### Client behaviours inside and outside sessions

In this subtheme, participants described the way the therapy effected their behaviour in and out of sessions. This included aspects such as engaging in greater assessment of their situation, selecting their behaviour according to consequences, doing more positive things, a change in the pattern of their behaviour towards others including becoming more likely to accept help and being more open with others about their condition and responding differently to mood state.

##### More assessment of the situation

In some cases, participants described becoming more able to analyse life situations to understand the best way to approach them. This could include considering the ‘big picture’ in terms of the participant's own goals and how these could guide one's behaviour in the situation.

Ummm, I think just analyzing the situations more or preparing myself better for the situations (Clara).

Whereas the sessions, ummm, encourage you to take a step back and think about the big picture.

And ok you don't feel great now, but where do you wanna be? And its about refocusing ummm, yourself to that goal and the choices that you make (Jennifer).

##### Selecting their behaviour according to consequences

A number of participants said that therapy allowed them to make a choice about their behaviour based on its consequences. This led to them become more conscious about their actions and behaviours around other people.

So I went running with some friends of mine on a Monday night or Wednesday night. For a while I was doing Monday and Wednesday, so I'm planning to do tomorrow as well. So it was just nice, I guess that had helped exploring what helps and seeing friends help and doing exercise helps (Gemma).

##### Doing more positive things

Some participants reported engaging with positive activities more, which led to more positive feelings.

And I was remembering this horrible feeling I have that I just do not wanna do anything. I can feel it now, and it is not an actual feeling I have but I remember what it's like. So the difference now is I am actually out and about and I am actually seeing things and like smelling things. I am actually like enjoying itand appreciating and thinking of the future (Jennifer).

##### Changing pattern of behaviour towards others

Behavioural change towards others was considered important by the majority of participants. One example that was described by several participants was that of changing a pattern of helping others, which had previously been to the detriment of their own well‐being.

Yeah focusing on my well I guess my wellbeing holistically rather than… It is very much I would say put the gas mask on first before you helped someone else because you're not going to be in a good place (Jennifer).

A subtheme of note within ‘changing behaviour towards others’ was the reported tendency to become more open to others about the condition and more open with regard to seeking help.

Yes. There's been—I'm actually able to accept people helping, that it's not a criticism on me. And I don't have to sit there and beat myself up, that it's because somebody thinks I can't do it (Judy).

I found helpful from the study was that actually being a bit more open with people about my diagnosis and my condition has helped. And I'm still being quite cautious about that but that has been where I've told people its been quite positive, that was useful to explore in the sessions as‐well. That was helpful so whereas previously I might not have said anything erm yeah that's been helpful (Gemma).

##### Responding differently to mood states

The majority of participants expressed that therapy changed how they responded to mood states, other people, thoughts and situations. Below, one participant describes responding differently to the realities of her physical health situation and implies that this represents a change to a habitual pattern of responding and reduced self criticisizm.

My biggest one is that whole like letting things go. And not sitting there blaming myself. An example is I recently had chest infection so obviously doing things at home hasn't, hasn't got done. Whereas normally in the past, I would literally have crawled myself around doing it because then it would have to be done because I hadn't been able to do it I then start nagging at myself in my head and like, how bad it was, how pathetic it was, how much blah blah whereas I've been able to let it go a little bit and although I've still been like ‘oh I should have done it’ it's been a ‘oh I should have done it’ rather than a beating myself up about it. So it's making steps in the right direction, definitely (Judy).

Learning how to respond differently to mood states was seen as improving mood stability. For example, one participant described how therapy enabled her to respond differently to high mood.

Because when you feel high you just wanna go, go wild, go out, and just go flirting and you know. Ummm I think by going ok I don't need to do that behaviour, I don't actually want to and I know what the impact of that has on my life, my values, my relationship, ummm. So actually if I do this other thing, I'm still going to have fun but I am not going to do any of those things that ruin my values (Jennifer).

#### Changes in client perspective

The participants discussed the factors that contributed to a change in their perspective as part of this subtheme. This included choosing to see the situation differently, increased acceptance and changing beliefs.

##### Choosing to see the situation differently

Some participants reported finding the therapy helped them better focus on what was happening around them and selecting the positive aspects of what was going on, which resulted in changing their mindset.

Being able to let go of some things from the past and trying to let go of some unhelpful rumination you know and trying to focus you know, usual mindfulness, you know focus more on what is happening you know today (Becky).

Because quite a lot's gone on recently, just sort of trying to you know, really focus on the positives you know trying look at things differently (Clara).

##### Acceptance

It appeared that therapy helped some participants to gain a sense of acceptance; this included acceptance of mood ‘ups and downs’, acceptance of the self and acceptance of the condition, which helped participants to feel more stable. Although acceptance could be considered to be a form of belief change, in the analysis we coded this as a separate theme to ‘change in beliefs’ because of its prominence and also because acceptance implies both a change in perspective and a change in how one orients oneself towards a situation and feels disposed to act in relation to it, which was very much in keeping with what our participants described.

I remember that was particularly helpful with was guess kind of accept some of the ups and downs where previously I would try and fight them; if this is low, this is terrible; I've got to do something about it (Gemma).

I accept that I am not going to be what I normally used to feel like, but I am a little bit closer to that and I can live a bit of a better life now (Jennifer).

##### Changing beliefs

It was noted by many participants that therapy helped them to change their perspective. This could include a new perspective on a pattern of behaviour, on one's personal values, on the self, or on the condition itself.

(Deep sigh) … I think the awareness that I came to that I did not have to be the life and soul of the party. That I did not have to be the entertainer. That I did not have to be one that was responsible for whether everyone else in the room was having a wonderful time or not, emm, was probably pivotal in me being able to head, head off the the high mood at the pass so to speak (Lisa).

#### The impact on symptoms

As a part of this subtheme both the immediate impact of the session and the medium‐term impact of the therapy on mood were described by the participants.

##### Effects of the sessions in the short term

Some participants described the immediate impact of therapy sessions on their mood as distinct from the longer term, cumulative impact.

I remember when I first had my session, I left and I just felt over the moon. I was like this is perfect for me. Like everything we spoke about was … it really gave me a boost. And I thought yeah, this is going to be good (Jennifer).

##### Effects of the therapy on mood in the medium term

Alongside this short‐term effect, some participants also emphasized that therapy had some impacts on mood in the medium term. In particular, the emphasis was often upon stabilization of mood.

Sometimes I won't be able to control it but if I put this in place and that in place and do this and do that. I can kind of almost ground myself to kind of almost be a bit more stable. And if that can control it, amazing. And if it can't at least I put in everything that I could possibly do that I have learned to be able to stay (Yvonne).

#### Impact on life and functioning

Within the scope of this subtheme, participants talked about how therapy had impacted upon their functioning and their lives more generally. This included improved relationships and changes in engagement in life.

##### Improved relationships

Some participants discussed the impact on relationships with family and friends: therapy was seen as bringing about positive changes in this domain through changes in the client's behaviour in relation to others.

There has been a big movement in my relationship with [family members] so you know all of that has moved quite considerably. I am not sure it has moved in a positive way but it has certainly moved in a way that's very positive for me. You know I no longer I feel I need to take responsibility for all of that. You know that that really, really made a big difference (Lisa).

##### Engagement in life

Another aspect of this subtheme was how therapy impacted the participant's engagement in activities. One participant highlighted that as a result of the therapy, their engagement with some activities has increased.

I think at work I am very much, I am very aware, I'm more aware of what is going on and I want to get involved and I want to do good things. Ummm, and I guess with other people I am more cautious of the way I respond to them and I think this is where the question is? Yeah (laughs). Ummm, yeah, I guess I just feel more positive day to day. So that I want to go out and do more things (Jennifer).

#### Thematic synthesis

**FIGURE 2:**
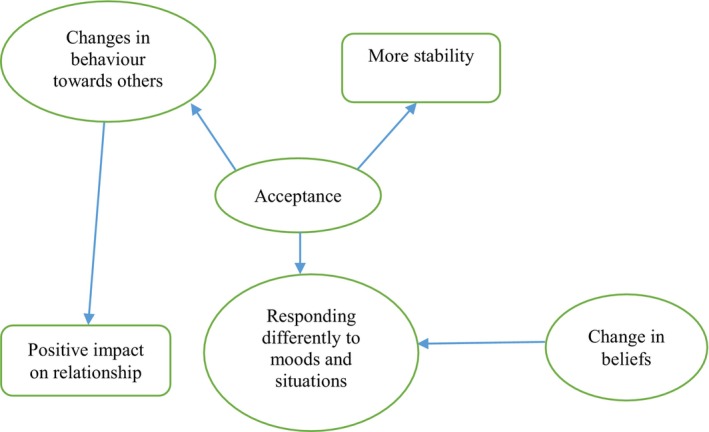
Schematic depicting interrelationships between themes according to participant accounts. Oval boxes: Process of change; rectangle boxes: Outcomes.

Figure 2 depicts a summary of the interrelationships between subthemes as described by participants. Subthemes are included only when their relationship to another subtheme was explicitly mentioned by at least one participant; arrows connecting subthemes are included only when this relationship was mentioned by at least one participant.

Changes in acceptance were seen as leading to: more stability, being able to respond differently to moods and situations and behavioural changes in interactions with others. Behavioural changes in interactions with others were in turn described as positively affecting relationships, whilst being able to respond differently to moods and situations was described as being contributed to by a change in beliefs as well as greater acceptance.

## DISCUSSION

In this study, the aim was to explore participant's perceptions of the process of change within behavioural activation adapted for bipolar depression and of the impact of the therapy both during and after the therapy period.

In line with what would be expected from the theory of change underpinning behavioural activation (Kaiser et al., 2016) behaviour change was described by many. This was particularly in terms of changes in patterns of behaviour towards others and changes in behavioural responses to specific situations and mood. Several individuals reported that as a result of therapy, their engagement in positive activities increased. This is consistent with a 2017 study in which BA delivered by junior mental health workers was compared to cognitive behaviour therapy delivered by professional psychotherapists in terms of acceptability, mechanisms of change and impact (Finning et al., 2017). Also consistent with the findings of Finning and colleagues, our participants reported changes in unhelpful behaviour patterns and the facilitation of this through increased awareness of consequences.

Participants regarded behavioural change as an important aspect of the treatment programme and because of its conceptual orientation and treatment objectives, this is not surprising. There was, however, also a description of what might be viewed as cognitive change among those receiving treatment. Acceptance of the self and of the condition, as well as embracing ‘ups and downs’, was a key theme across participant accounts, and from examination of participant descriptions of the interrelationships between subthemes was reported to be a central factor in facilitating behaviour change. The results were similar to those of Joyce et al. (2017) who examined people's experiences with a novel cognitive behavioural therapy for bipolar disorder and Poole et al. (2015) who examined the perspectives of patients in relation to group psychoeducation for bipolar disorder in detail in which participants reported greater acceptance of their conditions. Therapy bringing about a change in beliefs was also reported by participants in this study, however from their accounts of the interrelationship between sub‐themes, this was described as playing a less central role than was acceptance. Acceptance has been characterized in the literature as an important component of both clinical and personal recovery for people with bipolar disorder (Chirio‐Espitalier et al., 2022). In a qualitative study with individuals with bipolar disorder using a grounded theory approach Pereira et al. (2024) proposed a central role for acceptance of the diagnosis and condition in faciltating an individual and non‐linear recovery process. Our study complements their suggestion that acceptance may promote the use of new coping strategies and engagement in social roles, thus contributing to personal recovery.

The central role of acceptance may be considered an unexpected finding: traditionally, behavioural therapy for depression places emphasis on behaviour change and though those may facilitate changes in cognitive appraisals, the latter are not targeted directly. Therefore, it might be expected that behaviour change would be reported as preceding and promoting changes in acceptance. Because the therapists had been recently involved in a DBT adaptation, some of the principles or techniques from DBT may have been brought into the therapy protocol being studied, which could have had an impact on the observed results regarding the importance of acceptance in the therapeutic process. Conversely, in order for a patient to be willing to engage in behaviour change, they need to be sufficiently motivated to do so. The findings suggest that increased acceptance of the self and one's tendency towards high and low moods may, for some people, be an important precondition of behaviour change, which then helps bring about important longer term outcomes such as improvements in relationships and mood stability. Although not explicitly targeted within behavioural activation, increased acceptance of internal states is a target of some other therapy approaches such as Acceptance and Commitment Therapy (ACT: Hayes et al., 2012), Mindfulness Based Cognitive Therapy (MBCT: Segal et al., 2002) and Dialectical Behaviour Therapy (DBT: Dimeff & Linehan, 2001). Indeed, some of the current findings, including responding differently and a greater acceptance of mood changes, are also reported in an analysis of participants' accounts of receiving MBCT for BD (Chadwick et al., 2011). Taken together, this raises the possibility that the impact of behavioural activation for bipolar depression could be increased by harnessing concepts and techniques from these approaches.

In terms of the overall impact of therapy, it is notable that some participants in our study described therapy as helping them become more stable. This is, however, in contrast to the findings of Finning and colleagues, whose participants described therapy as leading to mood improvement rather than mood stability. This may reflect subtle differences between studies in the priorities of either patients or therapists leading to an increased focus on one outcome versus the other.

### Strengths and limitations

To enhance the dependability of our findings (Lincoln & Guba, 1985) we adopted a rigorous approach to data analysis. Two researchers independently reviewed and coded the transcripts, with ongoing discussions and input from a third researcher to refine the coding framework. Subsequent coding of the transcripts within the framework and thematic analysis were further validated by having a second researcher verify the coding and analysis and resolve discrepancies. Throughout the process, original quotations, grouped by subtheme within the charting document, were referred to in order to enhance confirmability; also in service of this, the two coders generated reflexive statements.

With regard to the credibility of our findings, we were able to interview the majority of those who received the therapy, and interviews were conducted by researchers who were not involved in therapy delivery in order to reduce demand effects.

One of the main limitations of the study is that although the case series study included three male and nine female participants, we were able to interview the female participants only. There may have been divergent opinions among the participants who declined to participate in the qualitative study, and this may be exacerbated by the gender difference between those who did and did not participate. Similarly, all participants were white British and living in the south west of England, potentially reducing the transferability of our findings.

Another limitation is that some of the interviews were conducted via phone as a result of the COVID‐19. It is possible that over the phone, there may be a loss of contextual and nonverbal information since there are no visual cues to guide the conversation (Novick, 2008). The protocol was also delivered during COVID‐19, potentially limiting the opportunities for activation.

Our synthesis of the interrelationships between subthemes (represented by Figure 2) cannot be considered definitive, even within our small group of participants, as we included connections between subthemes only when these were explicitly mentioned in order to enhance credibility. It is possible that participants experienced other interrelationships but did not comment on these during the interview. Similarly, no single participant described all of the interrelationships depicted in Figure 2. As such, this represents a compilation of accounts, and it is possible that the entire process depicted would not occur for any single individual.

As with other qualitative studies, caution should be exercised in generalizing conclusions beyond the sample; instead our findings suggest avenues for further exploration. We are also limited in our ability to compare our findings directly with those from similar studies in other populations (such as the study by Finning and colleagues) because of differences in setting, interview topic guide, interviewer and analysts.

### Implications

Our findings tentatively suggest that the theory of change underpinning behavioural activation for unipolar depression may be broadly applicable when a similar protocol is used with people with bipolar depression. Alongside this, the potential for increased sense of acceptance (of the self and of mood states) to play a role in facilitating behaviour change could be explored further with regard to its place within a theory of change. This could be done as part of further examination of the process of change within behavioural activation for bipolar depression, using both quantitative and qualitative methods within clinical trials. The processes themselves may be measured using traditional mediation designs; however, more novel methods such as experience sampling could be used to permit examination of the interaction between feeling states and behaviours over time (Myin‐Germeys et al., 2018).

Clinically, our findings tentatively suggest that therapists delivering behavioural activation to individuals with bipolar depression might seek to enhance their patient's sense of acceptance (for example, of their internal states) in order to facilitate behavioural change. This could involve borrowing concepts and techniques from related approaches and deploying these within the broad behavioural framework of the therapy.

## CONCLUSIONS

Our study is the first to explore participants' experiences of BA in the treatment of bipolar depression using a qualitative approach. Our findings suggest avenues for further investigation in the development of the underpinning theory of change. To enable this, future studies of clinical efficacy might consider including measures of key processes described by our participants, as well as those clearly indicated by the behavioural theory of depression.

## AUTHOR CONTRIBUTIONS

**Sakir Yilmaz:** Conceptualization; formal analysis; investigation; methodology; project administration; writing – original draft; writing – review and editing. **Anna Hancox:** Formal analysis; investigation; writing – review and editing. **Molly Price:** Investigation; writing – review and editing. **Jemma Regan:** Investigation; writing – review and editing. **Barney Dunn:** Conceptualization; writing – review and editing. **Heather O'Mahen:** Conceptualization; writing – review and editing. **Kim Wright:** Conceptualization; formal analysis; investigation; methodology; project administration; supervision; writing – review and editing.

## CONFLICT OF INTEREST STATEMENT

All authors declare no conflict of interest.

## Supporting information


Data S1.

Data S2.

Data S3.

Data S4.

## Data Availability

To protect the anonymity of participants, access to transcripts of the qualitative interviews is restricted to members of the research team.
