# Supplementary material for: Patient experiences of behavioural therapy for bipolar depression: A qualitative study
Source: Br J Clin Psychol. 2024 Dec 10;64(3):553–68. doi: 10.1111/bjc.12515 (PMC12334977; doi:10.1111/bjc.12515)
Supplement: Supplementary file 1 — Data S1. [file BJC-64-553-s001.docx]

**Table X**

*Interview topic guide*

| **Topic of discussion** | **Probe areas** |
| --- | --- |
| **An overview of the treatment experience**  How did you find the therapy? Can you describe your experience? | - Did you experience what you expected from BA-BD therapy?   - Most helpful things about the program   - Anything that was not particularly helpful - Did the BA-BD program surprise you in any way? |
| **Delivery of Treatment**  Several components made up the therapy: one-on-one sessions, handouts and a smartphone application. Could you tell me what you thought of these? | - Comfortable with your therapist  - Your therapist`s understanding  - How much you felt able to trust your therapist  - Views on number of therapy sessions  - Views on frequency of the therapy sessions  - Experience of booster therapy sessions |
| **Impact of Treatment**  What effects has the programme had for you? | - Noticing any changes in your mood or the pattern of your mood   - Any changes in your thinking  - Any changes to your high moods  - Any difference in how you respond to your moods  - Any differences in how you approach their everyday life  - Any differences in your patterns of behaviour  - Any activities or things you do less or more of now  - Any differences in how you interact with other people  - Any impact on your relationships |
| **Barriers to Treatment**  It is important for us to understand what motivates or prevents people from fully engaging with BA-BD. | - how many BA-BD sessions attended  - What influenced how many BA-BD sessions you attended?  - How much homework completed  - Timing of the BA-BD sessions  - Length of the BA-BD sessions  - location of the BA-BD sessions |
| **Experiences of the Research Project**  What was it like taking part in the research study? | - Completing the weekly questionnaire measures - Completing the questionnaire packs - Completing the interviews - The volume of questionnaires - Contacting with the research team - Anything that the researchers could do differently |
| *Note.* BA-BD: Behavioural Activation Therapy for Bipolar Depression | |
