# Supplementary material for: Patient experiences of behavioural therapy for bipolar depression: A qualitative study
Source: Br J Clin Psychol. 2024 Dec 10;64(3):553–68. doi: 10.1111/bjc.12515 (PMC12334977; doi:10.1111/bjc.12515)
Supplement: Supplementary file 2 — Data S2. [file BJC-64-553-s004.docx]

Acceptability of therapy

Within this theme participants talked about what they liked and disliked about the therapy. We identified five subthemes that describe participants' opinions on the acceptability of therapy: structural aspects of therapy, information delivery, therapeutic stance and relationship, helpful techniques and the context in which therapy occurs.

Structural aspects of therapy

In this subtheme, participants talked about aspects of the way the therapy was constructed that helped or hindered the effectiveness of the therapy. This included aspects such as the structured nature of the intervention, flexibility in contact and delivery, strengthening and repetition within the therapy, and the proactive nature of the approach.

*Structured nature of the intervention*

Many participants found the regularity of the weekly sessions helpful. Several participants also described the timing and the length of sessions as being ideal. The benefit of structure within the therapy content was also remarked upon by several participants, who commented that the presence of goals set at the beginning of therapy provided a context and something to work toward. In the following quote, Jennifer also expresses appreciation for a structured view of the week ahead at the end of each session.

She [therapist] set expectations. Ummm, so it was very clear what was gonna happen and ummm, that’s

probably why I felt so good after the first session when I left because I knew exactly what we were

gonna do and just felt positive. And ending the sessions, yeah it was, ummm, always thinking ok

let’s think about the week ahead, what challenges might come up and how can you make sure that

you’ve put everything you need and you’re assessing the risk in the week. So yeah (**Jennifer**).

*Flexibility*

Within this structure, flexibility was appreciated by the majority of participants in terms of flexibility of contacting the therapist. For example, being able to talk to them on the phone if feeling unable to come in to the therapy office, or the flexibility of delivery method of the therapy.

At the beginning we had, and it wasn’t like I found it helpful, we had like diaries that you would

for so many sessions keep a diary. I found them really difficult to, ummm, kind of work. Ummm,

some of them didn’t, because it was I think it was hour by hour one which is insane. Nobody can

really do that. And the layout was all umm, It just did not so much sense and I kind of like designed

a little one and gave to [Therapist]. This is kind of what works for me. She was like you know if it works

for you great, continue to use it (**Yvonne**).

A consequence of this flexibility noted by one participant was that it led to the therapy feeling "natural" and individualised.

Yeah I mean I have had therapy sessions before where it felt a bit like person was just following … it

just did not feel very natural. That’s only one time that’s happened. Maybe the person was a bit newer

to it. But with [therapist] it just felt very natural, very, you know, I did not feel like I was being processed

or yeah (**Jennifer**).

*Strengthening and repetition*

Various aspects of the therapy were mentioned that strengthened learning. One of this was repetition of concepts. Some participants highlighted the way that the therapy was organised to allow for repetition that helped learning as this brings a long-term solution and a guide for life instead of only trying to solve the problem right now.

I don’t know, but like you know when you have got to do something so many times to sink in.

Because it was probably only the last maybe six seven sessions that I was finally learning. It takes

quite a while to do something you have never done before. And ok 12 weeks sounds like a lot but

it is an hour (laugh) and you got to try and change the the biggest thing. So I think any less it wouldn’t

sink in. So I think yeah about the same and with the option of the booster sessions just cements it even

more (**Yvonne**).

Another aspect of therapy delivery that strengthened the learning was the option of booster sessions. This is because it was seen particularly helpful element of therapy by several participants as it was perceived as safety net. In other words, it provided the sense that support had not been withdrawn completely. Another reason that it was seen as useful is that booster sessions helped identify the areas in which triggers occur in life and reinforced the habit of self-awareness and self-reflection.

These booster sessions I come in and I am like this is going on and I can`t cope so then she says

right in these twelve weeks we have learned this. You tell me what you can use for this scenario.

Ummm, so she brings it all back out again which then cements it. Because I am ok like I know it I

have learned it. Ummm, yeah so I think those booster sections are vital because it just reminds you

(**Yvonne**).

*Proactive*

Some participants described the therapy approach as being proactive and found this helpful as it helped them to take action.

Uumm, I don’t know quite what to expect erm, I think I, I guess the one thing… that was

slightly different from an expectation maybe was that erm, but also quite obvious it was kind

of more about taking action than talking about how I felt so erm, err……so yeah yeah that’s

yeah probably yeah (**Gemma**).

Information delivery

This subtheme included aspects such as guided discovery/learning from my own experience; individualization; expressing and exploring.

*Guided discovery/Learning from my own experience*

Many participants highlighted the importance of guided discovery and learning from their own experiences. Particularly in terms of guided discovery, in order to assist them in solving their problems, alternative perspectives and solutions were found together. In the subsequent quote, Lisa emphasized that therapist support and guidance is essential to find a solution to their problems.

And you know these little tricks all the time that I was coming up with or she was coming up with

or between us we were coming up with that were really helpful. And I think she probably had a a raft of

them up and down her arm that a part of the of the whole process the the but they were sort of revealed to

me in not not through her suggestion but by my sort of arriving at them (**Lisa**).

*Individualisation*

Participants expressed appreciation for the individualisation of therapy content within the sessions. This included helping participants make strategies and techniques their own, and working out together what would work best for them.

Ummm, I think in cbt you are not actually allowed to talk about stuff. If you know what I mean,

it is very much you only talk about the process. And then if you deviate slightly to what is going in your

life, they steer right (laughs) right back to cbt whereas I think with this therapy even though they steer

you back to what you’re doing, you’re allowed to sort of relate it to your life more than, ummm, cbt

(**Becky**).

For one participant, individualisation also took the form of sharing metaphors that worked for her.

I think I what I came to realize through this was that I did not have to I did not have to go

that extra I did not have to just well I think before I have never really thought I could stop it. Was just

was just how it was. You know that’s where you got to and once you were up you are moving so fast

you couldn’t change gear down you just you just going and and I have learned that I have got a gear

stick now that I can I can you know owww ok (**Lisa**).

*Expressing and exploring*

There were a number of participants who reported that it was helpful to speak to someone and explore issues.

Ummm, because I have been going through some, quite some difficult times and it has just

really been good to have therapy and to be able to speak to someone the thing that was going on

during that time (**Clara**).

However, one participant emphasized that whilst exploration was helpful, as her mood cycle is very entrenched, it would be unlikely to have any impact on it.

Well like I say I’m not sure it’s going to have any impact on my mood cycle because it is very

entrenched, but like I say it’s been helpful to explore issues (**Lori**).

Therapeutic stance and relationship

In order to clarify the role of the therapist within this subtheme, participants described perceptions of the role of the therapist. A supportive therapeutic relationship between the therapist and the client was seen as very important for the success of treatment. The example of this included the therapist showing concern and holding the patience in mind, which encouraged participants to persevere in therapy.

Yeah. That whole thing. Having somebody actually on the other end of the phone or being

able to reach out – we had one session where my questionnaire results were alarming, shall we say,

and I was contacted by [therapist] just as a check in which was really good (**Judy**).

This also included the style of the therapist adopted. There was a strong emphasis among a number of participants on the importance of the therapist adopting a non-judgemental attitude during the therapeutic process, which particularly helped participants to become more open to seeking assistance.

I think she understood me very well. I think you know because I was able to be very candid I

think she had came to quite a good understanding of the sort of person I was. And and she was

completely non-judgmental which is is a rare thing in today’s world. I mean I know that it is her

professional responsibility but it was appreciated (**Lisa**).

Potentially related to this non-judgmental approach, two participants reported not feeling under pressure from their therapist about their progress due to the fact that they could stop therapy at any time and the therapist was not pushing them to follow any particular course of action and this was seen as helpful.

Yeah yeah yeah I would bring it every week. Umm, It was never pressure from [therapist], like you

need to do that, you need to do that. Ummm, but it is, and you know somethings worked and

somethings did not. And yeah there can be a lot (**Yvonne**).

Alongside this, participants also expressed their appreciation for the encouragement offered by the therapist, for example in making changes, which helped keeping participants on track and to achieve some small steps.

Yes it (therapy) was encouraging when I was struggling (**Tamsin**).

Ummm, it was good to talk in an open, confidential kind of way, exploring some of the issues that I had.

And you know I kind of respond well to that one-to-one work and it was good having someone specific

that kind of kept me on track and it kind of helped me to look at some small steps I was encouraged to

take to achieve (**Lori**).

Several participants expressed an appreciation for what a crucial role trust plays in forming a good therapeutic relationship, and noted it as an important factor in their therapeutic relationship.

Yes I felt I can trust her, yeah. I felt she knew what she was doing and ummm, she is very

sort of, ummm, professional but, ummm, very warm (**Becky**).

The presence of concrete techniques

The provision of concrete and specific techniques and strategies was identified as helpful by a number of participants. Participants particularly expressed how these techniques gave them confidence that they could handle difficult situations and mood states differently.

Ummm, I think it sort of gives you techniques which you can then incorporate into your daily life,

ummm, not perfectly, obviously, because, but they are, they do sort of stay with you even if they

do not initially come into your mind after you know you start think oh maybe I can handle it differently,

ummm, or, ummm, not that it’s easy (**Becky**).

In addition to this, one participant stated that the techniques had helped them deal with the mood swing difficulties that she had faced in the past, should they recur.

It works for me because I have been given concrete mental/ physical tools for dealing with

previous difficulties in mood swings particularly (**Lisa**).

Context in which therapy occurs

In this subtheme, participants talked about the impact of the context in which the therapy took place. This included not only the treatment context in terms of their care pathway but also their family and personal context.

*Relationship to medication*

In terms of the treatment context, a number of participants considered therapy in relation to pharmacological or psychological treatment they were receiving currently or had received in the past. In some instances, psychological therapy was seen as adjunctive to the medication.

Yeah I think the therapy is just 100%, no 95% (laughs) and then - yeah I think more than that –

yeah I think 95 and then the medication just gave me that little something that I needed and helped me

through (**Jennifer**).

Another participant describes therapy as preferable to medication because medication is not seen as addressing the root of the problem.

Then - not just that, what I would say to her, but how I would cope and how I would deal with

those breaking it down and dealing with each thing, ummm, rather than just taking another tablet or

forgetting it or suppressing it or not dealing with it (**Yvonne**).

*Person`s family context*

A small number of participants mentioned the attitude of their families towards to therapy as a facilitating factor.

My friends and family weren’t questioning or undermining it so that was good, so in fact I think

um my husband was quite encouraging so yeah it didn’t adversely affect it (**Gemma**).

Barriers to therapy

A number of participants mentioned the forms or questionnaires that they were invited to complete as part of the therapy. Aspects of these that were seen by some participants as problematic included the amount and being uncomfortable. Several participants commented that the amount of paperwork involved in the process was too much for them.

Hour by hour sheet is too much. Probably right in the beginning when [therapist] gave me a

sheet sort of every hour how you were feeling and what you were doing, I just found that a

bit too much. For me it was too much in my head to think about. As you’ve probably guessed I’ve

got a bit of a goldfish brain so trying to remember to do stuff every hour was, yeah it doesn’t

work (**Judy**).

Participants were asked to complete brief weekly measures as part of the therapy and also as part of the research process. One participant commented upon discomfort whilst filling out the forms when unwell because it made her confront those feelings. At the same time it was acknowledged that the forms are simply confirming what she already knew.

I guess it felt sometimes pretty disappointing to be kind of like filling in when your not feeling as

good but I think that’s just because you’re just writing what you know already so it’s kind of reflected

back at you but yeah I didn’t mind doing it (**Gemma**).

Discussion

There were several important factors highlighted by participants that made therapy acceptable. The establishment of goals at the beginning of therapy constitutes one of the important structural aspects of psychotherapy which has not been mentioned in other qualitative studies of participants with bipolar who have received psychological therapy other than BA (e.g. Mansell et al., 2010; Poole et al., 2015). Some participants considered it quite important for the therapy to be flexible in its delivery method. This flexibility led to a feeling of "naturalness" and individualization in the therapy, as noted by one participant. Participants reported that the repetition of concepts contributed to learning since it led to a long-term solution instead of just solving the problem in the present. Repetition of concepts is a key aspect of the delivery of BA however other qualitative studies of BA for UD (e.g. Finning et al., 2017) do not report mention of this by participants. In addition, this finding is consistent with that of Harvey's (2016) study, which tested a novel memory support intervention in an attempt to improve patient memory for treatment and treatment outcomes. They found that memory support may help patients remember their treatments better and may even lead to better results. The offering of booster sessions was another facet of therapy delivery that enhanced the learning process. Whisman (1990) study showed that the preservation of treatment-induced behavioral changes has proven somewhat successful with booster maintenance sessions.

In the current study learning from one's own experiences and guided discovery were important to many participants. A therapist's approval and guidance are essential to finding a solution, particularly in guided discovery. Learning from experience (experiential learning) is an essential component of BA while guided discovery is traditionally a CBT concept, however, participants expressed their appreciation for this being used in BA delivery. There was a sense of individualization in therapy which means in addition to addressing current issues, its delivery was tailored to the needs of participants. In keeping with the findings of Finning et al. (2017) and Straarup and Poulsen (2015) who conducted qualitative interviews with participants with unipolar depression who received similar therapies, a positive therapeutic relationship was found to be important as it assisted participants in becoming more open to seeking help and supported participants' perseverance in therapy. Numerous meta-analyses (e.g., Flückiger et al., 2012) have demonstrated the robustness of the statistical link between alliance and result. Participants also identified concrete and specific techniques and strategies as helpful because they provided confidence that things can be handled differently. As such, this underscores the importance, as discussed in BA, of being concrete and specific with patients when discussing their difficulties and how to address them. A majority of these findings (including therapeutic stance and relationship, individualization, flexibility, specific techniques and metaphors) are consistent with reports from participants of a qualitative study on the experiences of a novel CBT for BD (Joyce et al., 2017). Although this study shares some similar findings with our study, some aspects concerning the acceptability of the therapy, such as repetition of concepts, non-judgmental attitude of the therapist, and guided discovery, are unique to our study.

**References**

Finning, K., Richards, D. A., Moore, L., Ekers, D., McMillan, D., Farrand, P. A., O'Mahen, H. A., Watkins, E. R., Wright, K. A., Fletcher, E., Rhodes, S., Woodhouse, R., & Wray, F. (2017). Cost and outcome of behavioural activation versus cognitive behavioural therapy for depression (COBRA): a qualitative process evaluation. *BMJ open, 7* (4), e014161. https://doi.org/10.1136/bmjopen-2016-014161

Flückiger, C., Del Re, A. C., Wampold, B. E., Symonds, D., & Horvath, A. O. (2012). How central is the alliance in psychotherapy? A multilevel longitudinal meta-analysis. *Journal of counseling psychology, 59*(1), 10–17. https://doi.org/10.1037/a0025749

Harvey, A. G., Lee, J., Smith, R. L., Gumport, N. B., Hollon, S. D., Rabe-Hesketh, S., Hein, K., Dolsen, E. A., Haman, K. L., Kanady, J. C., Thompson, M. A., & Abrons, D. (2016). Improving outcome for mental disorders by enhancing memory for treatment. *Behaviour research and therapy, 81*, 35–46. <https://doi.org/10.1016/j.brat.2016.03.007>

Joyce, E., Tai, S., Gebbia, P., & Mansell, W. (2017). What are People's Experiences of a Novel Cognitive Behavioural Therapy for Bipolar Disorders? A Qualitative Investigation with Participants on the TEAMS Trial. *Clinical Psychology &Amp; Psychotherapy, 24* (3), 712-726. doi: 10.1002/cpp.2040

Mansell, W., Powell, S., Pedley, R., Thomas, N., & Jones, S. A. (2010). The process of recovery from bipolar I disorder: a qualitative analysis of personal accounts in relation to an integrative cognitive model. The British journal of clinical psychology, 49(Pt 2), 193–215. <https://doi.org/10.1348/014466509X451447>

Poole, R., Smith, D., & Simpson, S. (2015). Patients’ perspectives of the feasibility, acceptability and impact of a group-based psychoeducation programme for bipolar disorder: A qualitative analysis. *BMC Psychiatry, 15* (1). Available at: https://doi.org/10.1186/s12888-015-0556-0

Straarup, N. S., & Poulsen, S. (2015). Helpful aspects of metacognitive therapy and cognitive behaviour therapy for depression: A qualitative study. *The Cognitive Behaviour Therapist,* 8. Available at: https://doi.org/10.1017/s1754470x15000574

Whisman, M. A. (1990). The efficacy of booster maintenance sessions in behavior therapy: Review and methodological critique, *Clinical Psychology Review, 10* (2), pp. 155-170, <https://doi.org/10.1016/0272-7358(90)90055-F>.
