# Supplementary material for: Patient experiences of behavioural therapy for bipolar depression: A qualitative study
Source: Br J Clin Psychol. 2024 Dec 10;64(3):553–68. doi: 10.1111/bjc.12515 (PMC12334977; doi:10.1111/bjc.12515)
Supplement: Supplementary file 3 — Data S3. [file BJC-64-553-s002.docx]

Table X

Summary of themes and subthemes emerging from qualitative analysis

| Area of exploration | Themes and subthemes |
| --- | --- |
| 1. Acceptability of therapy | - 1. Structural aspects of therapy   1.1.1 Structured nature of the intervention  1.1.2 Flexibility  1.1.3 Strengthening and repetition  1.1.4 Proactive  1.2 Information delivery  1.2.1 Guided discovery/Learning from my own experience  1.2.2 Individualisation  1.2.3 Expressing and exploring  1.3 Therapeutic stance and relationship  1.4 Helpful techniques  1.5 The context in which therapy occurs. |
| 2. Impact of therapy | 2.1 Client behaviours inside and outside sessions  2.1.1 More assessment of the situation  2.1.2 Selecting their behaviour according to consequences  2.1.3 Doing more positive things  2.1.4 Changing pattern of behaviour towards others  2.1.5 Responding differently to mood states  2.2 Changes in client perspective  2.2.1 Choosing to see the situation differently  2.2.2 Increased acceptance  2.2.3 Changing beliefs.  2.3 The impact on symptoms  2.3.1 Effects of the sessions in the short term  2.3.2 Effects of the therapy on mood in the medium term  2.4 Impact on life and functioning  2.4.1 improved relationships  2.4.2 changes in engagement in life. |
