# Supplementary material for: Patient experiences of behavioural therapy for bipolar depression: A qualitative study
Source: Br J Clin Psychol. 2024 Dec 10;64(3):553–68. doi: 10.1111/bjc.12515 (PMC12334977; doi:10.1111/bjc.12515)
Supplement: Supplementary file 4 — Data S4. [file BJC-64-553-s003.docx]

I'm a middle-aged, white Turkish man in my 30s without any firsthand experience with bipolar disease. Since this was not my home country, I was conducting this study in a place where I had only lived for two years and was unfamiliar with the healthcare system and culture. Our team's collaborative approach, which included a different interviewer, coders, and transcript checkers from the UK, as well as a range of cultural viewpoints, was crucial in improving the scope and depth of our research. I've learnt to successfully negotiate cultural differences by appreciating the value of both insider and outsider perspectives and taking advantage of the distinctive insights they provide.

As a researcher with a background in psychology, my interest in the complexities of psychological practice and the management of mental illnesses was sparked during my time as an undergraduate. During my PhD studies, I became even more passionate about bipolar disorder and spent a lot of time conducting systematic reviews and meta-analyses to understand how effective psychological therapies are. In order to conduct systematic reviews, I had to carefully examine the literature and immerse myself in randomized controlled trials in order to understand the subtle differences in psychological treatments for bipolar disorder that are based on evidence. This difficult but illuminating journey gave me deep insights into the complex nature of bipolar disorder in addition to broadening my theoretical knowledge.

Through this procedure, I have gained a deep appreciation for the complexities inherent in treating this ailment. Moreover, my journey into qualitative inquiry has been profoundly enlightening and transformative. I have actively sought to embrace the ontological and epistemological principles underpinning qualitative research, recognizing that my predominantly quantitative background may influence my perspective in this exploration

As a non-clinician with no prior experience directly administering behavioral activation therapy, I approached the problem from a new angle when doing this research. There are benefits and drawbacks to this strategy. One way to look at things is from a different angle because my personal experience with therapy delivery does not influence my opinions. However, it was helpful to have a basic comprehension of the therapy in order to understand and evaluate the participant's observations during the interviews. I thoroughly researched behavioral activation and attended training sessions in order to get ready for this study, which enhanced my qualitative examination of the therapy's efficacy in treating bipolar disorder.

Oveall as a researcher with a strong background in psychology, my assumptions and beliefs may have an impact on my research. I therefore made an effort to keep a reflexive posture throughout the investigation, questioning my presumptions and prejudices and discussing my perspectives with other coders in the team.
